# The RhoJ-BAD signaling network: An Achilles’ heel for BRAF mutant melanomas

**DOI:** 10.1371/journal.pgen.1006913

**Published:** 2017-07-28

**Authors:** Rolando Ruiz, Sohail Jahid, Melissa Harris, Diego M. Marzese, Francisco Espitia, Priya Vasudeva, Chi-Fen Chen, Sebastien de Feraudy, Jie Wu, Daniel L. Gillen, Tatiana B. Krasieva, Bruce J. Tromberg, William J. Pavan, Dave S. Hoon, Anand K. Ganesan

**Affiliations:** 1 Department of Biological Chemistry, University of California, Irvine, Irvine, CA, United States of America; 2 Department of Dermatology, University of California, Irvine, Irvine, CA, United States of America; 3 Department of Biology, The University of Alabama at Birmingham, Birmingham, AL, United States of America; 4 Department of Translational Molecular Medicine, Division Molecular Oncology, John Wayne Cancer Institute (JWCI), Providence Saint John's Health Center, Santa Monica, CA, United States of America; 5 Department of Statistics, University of California, Irvine, Irvine, CA, United States of America; 6 Laser Microbeam and Medical Program, Beckman Laser Institute, University of California, Irvine, Irvine, CA, United States of America; 7 National Human Genome Research Institute, National Institute of Health, Bethesda, MD, United States of America; Stanford University School of Medicine, UNITED STATES

## Abstract

Genes and pathways that allow cells to cope with oncogene-induced stress represent selective cancer therapeutic targets that remain largely undiscovered. In this study, we identify a RhoJ signaling pathway that is a selective therapeutic target for BRAF mutant cells. RhoJ deletion in BRAF mutant melanocytes modulates the expression of the pro-apoptotic protein BAD as well as genes involved in cellular metabolism, impairing nevus formation, cellular transformation, and metastasis. Short-term treatment of nascent melanoma tumors with PAK inhibitors that block RhoJ signaling halts the growth of BRAF mutant melanoma tumors *in vivo* and induces apoptosis in melanoma cells *in vitro* via a BAD-dependent mechanism. As up to 50% of BRAF mutant human melanomas express high levels of RhoJ, these studies nominate the RhoJ-BAD signaling network as a therapeutic vulnerability for fledgling BRAF mutant human tumors.

## Author summary

BRAF^V600E^ is the most common mutation in human melanomas, and kinase inhibitors that block BRAF^V600E^ signaling can rapidly induce tumor regression but only modestly improve melanoma long-term survival. In this study, we identify a novel therapeutic vulnerability for BRAF mutant melanoma tumors. Targeting RhoJ signaling with existing PAK inhibitors was more efficient at blocking the progression of BRAF mutant tumors *in vivo* when compared to kinase inhibitors currently used to treat melanoma. Approximately half of all human BRAF mutant melanoma tumors express high levels of RhoJ, identifying the RhoJ-BAD pathway as a novel target for melanoma.

## Introduction

Oncogenes regulate cellular homeostasis in normal cells, and when mutated induce secondary physiological changes that stress cellular capacity for survival [1, 2]. Paradoxically, oncogenes drive the uncontrolled growth of tumor cells [3] and represent some of the most effective cancer therapeutic targets [4]. Unfortunately, inhibiting oncogene activity induces only short-lived tumor regression [5], eventually resulting in the regrowth of tumors that activate growth by other mechanisms [6–8]. Recent studies have sought to define pathways that allow tumor cells to cope with oncogene-induced stress [9]. Cancer cells are known to alter conventional signaling paradigms to skirt apoptotic stimuli [10]. In addition to avoiding apoptosis, tumor cells rewire metabolism to further facilitate growth [11]. An emerging approach to treat cancer is to identify non-mutated gene products critical for cancer cell and not normal cell survival and develop novel therapeutics to target these gene products [12]. These “non-oncogene” dependencies have proven effective drug targets for breast cancer and could potentially be used in other cancers such as melanoma [13].

The BRAF oncogene is the most commonly mutated gene in human cutaneous melanomas [14], and this oncogene also drives tumor cell proliferation [15]. BRAF mutations are not exclusive to tumors as they are also seen in common human nevi [16] that spontaneously arrest [2], and little is understood about what pathways allow BRAF mutant cells to proliferate to form nevi. Activating BRAF mutations and the loss of the tumor suppressor PTEN are events with a significant co-occurrence in melanoma [17]. These mutations result in the activation of MAPK and AKT signaling networks that accelerate melanoma development by promoting cell survival [18]. While MAPK and AKT signaling play an important role in melanoma progression, it is currently not clear what other pathways suppress oncogene-induced stress in BRAF mutant cells to allow them to proliferate.

RNAi approaches have been used to uncover selective tumor dependencies and identify novel therapeutic targets [19]. We previously used a genome-wide RNAi approach to identify RhoJ, a Cdc42 family GTPase, as a gene that allows melanoma cells to resist DNA damage stress *in vitro* [20]. RhoJ activates group I p21-activating kinases (PAK) in melanoma cells and PAK inhibitors can sensitize melanoma cells to DNA damage [20, 21]. In addition to modulating DNA damage stress, RhoJ modulates actin cytoskeletal dynamics in melanoma cells [21], and can regulate tumor angiogenesis in lung cancer xenografts [22]. Intriguingly, while RhoJ modulates multiple pathways that may be involved in melanoma growth, it is not mutated in melanoma tumors, suggesting that it may represent a “non-oncogene” dependence in tumor cells. In this study, we utilize physiologically-relevant *in vivo* systems to examine the role that RhoJ and its downstream targets PAK-BAD play in nevus formation and cellular transformation. We reveal that RhoJ modulates the growth properties and apoptotic threshold of BRAF mutant melanocytes, accelerating both the formation of nevi and the growth and metastasis of melanoma tumors. Surprisingly, treatment of mice with PAK inhibitors before tumors developed or during the early phases of tumor growth inhibits tumor growth and metastasis, nominating PAK inhibitors as a novel treatment for early-stage cutaneous melanomas.

## Results

### RhoJ regulates melanoma progression

**Fig 1:**
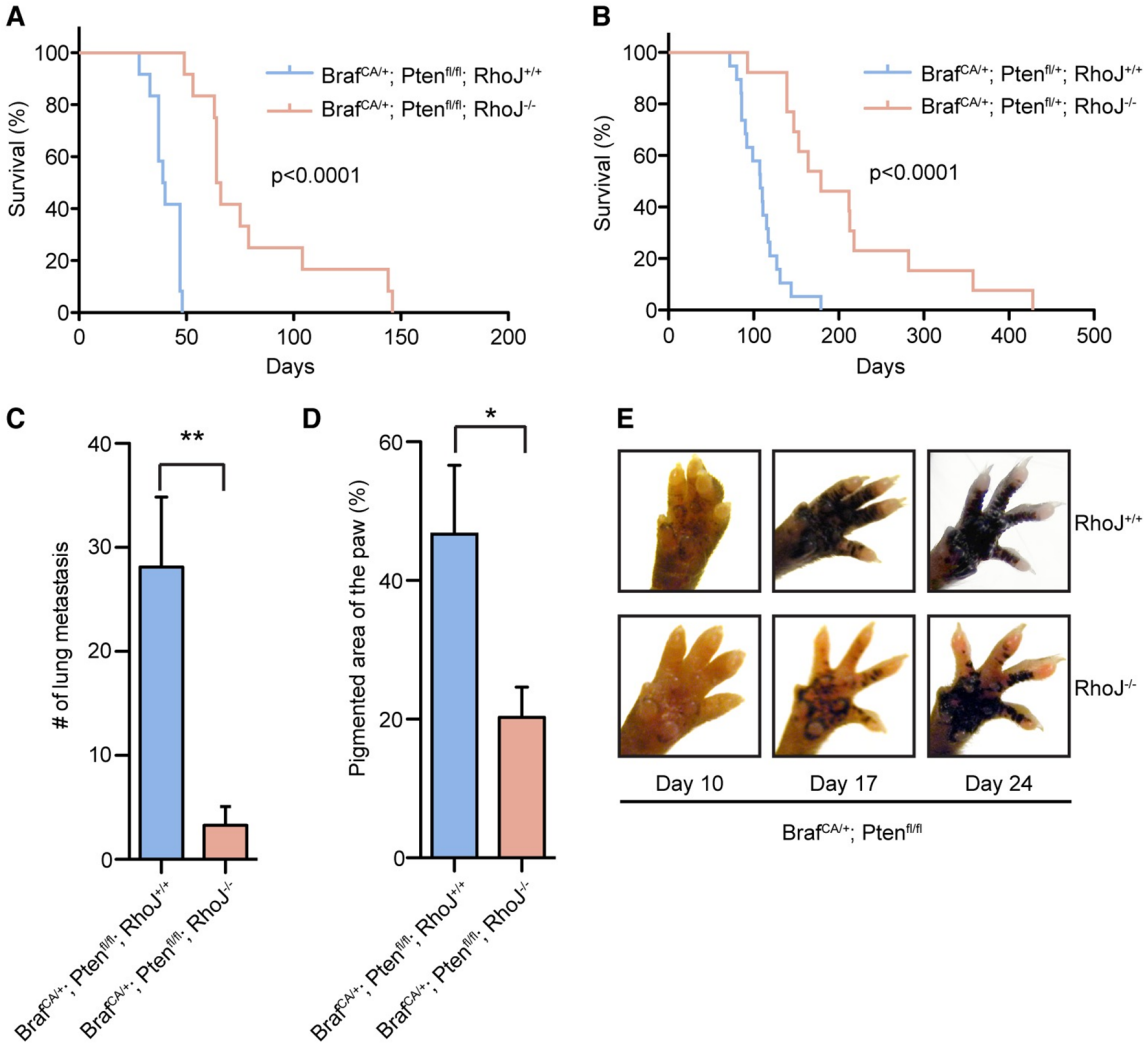
RhoJ regulates melanoma tumor development. (A) Loss of RhoJ prolongs survival of BRAF^V600E^ and PTEN null tumors. Kaplan-Meier survival analysis of 4-OHT treated *Braf*^*CA/+*^*; Pten*^*fl/fl*^*; Tyr*::*Cre*^*ERT2*^*; RhoJ*^*+/+*^ (n = 12) and *Braf*^*CA/+*^*; Pten*^*fl/fl*^*;Tyr*::*Cre*^*ERT2*^*; RhoJ*^*-/-*^ (n = 12) mice. Log-rank test demonstrates significant difference between *Braf*^*CA/+*^*; Pten*^*fl/fl*^*;Tyr*::*Cre*^*ERT2*^*; RhoJ*^*+/+*^ and *Braf*^*CA/+*^*; Pten*^*fl/fl*^*;Tyr*::*Cre*^*ERT2*^*; RhoJ*^*-/-*^ (p<0.0001). (B) RhoJ deletion prolongs the survival of mice carrying BRAF^V600E^ and PTEN haploinsufficient tumors. Kaplan-Meier survival curves of 4-OHT treated *Braf*^*CA/+*^*; Pten*^*fl/+*^*; Tyr*::*Cre*^*ERT2*^*; RhoJ*^*+/+*^ (n = 19), and *Braf*^*CA/+*^*; Pten*^*fl/+*^*; Tyr*::*Cre*^*ERT2*^*; RhoJ*^*-/-*^ (n = 13) mice. Log-rank test demonstrates significant difference between *Braf*^*CA/+*^*; Pten*^*fl/+*^*; Tyr*::*Cre*^*ERT2*^*; RhoJ*^*+/+*^ and *Braf*^*CA/+*^*; Pten*^*fl/+*^*; Tyr*::*Cre*^*ERT2*^*; RhoJ*^*-/-*^ (p<0.0001). (C) RhoJ deletion reduces lung metastasis. Lung metastases were compared in aged-matched (P30) mice using a dissection microscope (**p = 0.003, n = 8, Student’s t test). Error bars represent standard error of mean (SEM). (D) RhoJ deletion inhibits melanoma development. The amount of pigment present on the paws at day 17 was analyzed with ImageJ (*p = 0.04, n = 5, T-test). Error bars represent SEM. (E) RhoJ deletion delays melanoma progression. Paws of 4-OHT treated mice were imaged at 10, 17, and 24 days post birth.

To evaluate the role of RhoJ in melanoma development, constitutive RhoJ knockout (KO) mice were crossed with a previously described autochthonous mouse model of melanoma that carries a *Tyr*:*Cre*^*ERT2*^ allele, a *Braf*^*CA*^ allele, and one or two copies of a *Pten*^*lox4-5*^ allele [18] (S1A Fig). Initial studies validated that RhoJ knockout mice lacked RhoJ expression (S1B Fig). RhoJ deletion significantly inhibited the growth of melanoma tumors that expressed either no PTEN (p<0.0001, Log-rank; Fig 1A) or had one functional copy of PTEN (p<0.0001, Log-rank; Fig 1B), suggesting that RhoJ drives tumor growth by amplifying BRAF and not AKT signaling. Melanoma tumors from RhoJ KO mice metastasized to the lung at a lower rate as compared to RhoJ wild type (WT) mice (p = 0.003, Student’s t-test; Fig 1C, S1C Fig), suggesting that RhoJ regulates tumor progression, metastasis, or both. While the effects of RhoJ deletion on lung metastasis was dramatic, it was difficult to examine whether this phenotype was a consequence of a direct effect of RhoJ on lung metastasis or simply a consequence of the fact that RhoJ knockout mice had a lower tumor burden than RhoJ wild type mice at p30 (Fig 1C, S1C Fig). To determine whether RhoJ regulates tumor progression, tumors were induced on the paws of experimental mice and the kinetics of tumor growth was measured. There was a significant delay in tumor progression in RhoJ KO mice as compared to RhoJ WT mice. RhoJ KO mice developed early stage tumors (as measured by pigmented area) a week later than RhoJ WT tumors (Fig 1D and 1E, S1D Fig), indicating that RhoJ promotes the growth of developing melanoma tumors. When the paws of RhoJ KO mice were examined at a later timepoint (P33), we observed that the paws of the animals appeared similar to what was observed in RhoJ WT mice at p24 (S1E Fig), suggesting that RhoJ most likely affects either the initiation or progression of tumors and that RhoJ knockout mice likely have similar numbers of paw melanocytes as RhoJ wild type mice.

### RhoJ modulates signaling pathways that control melanocyte differentiation and survival

**Fig 2:**
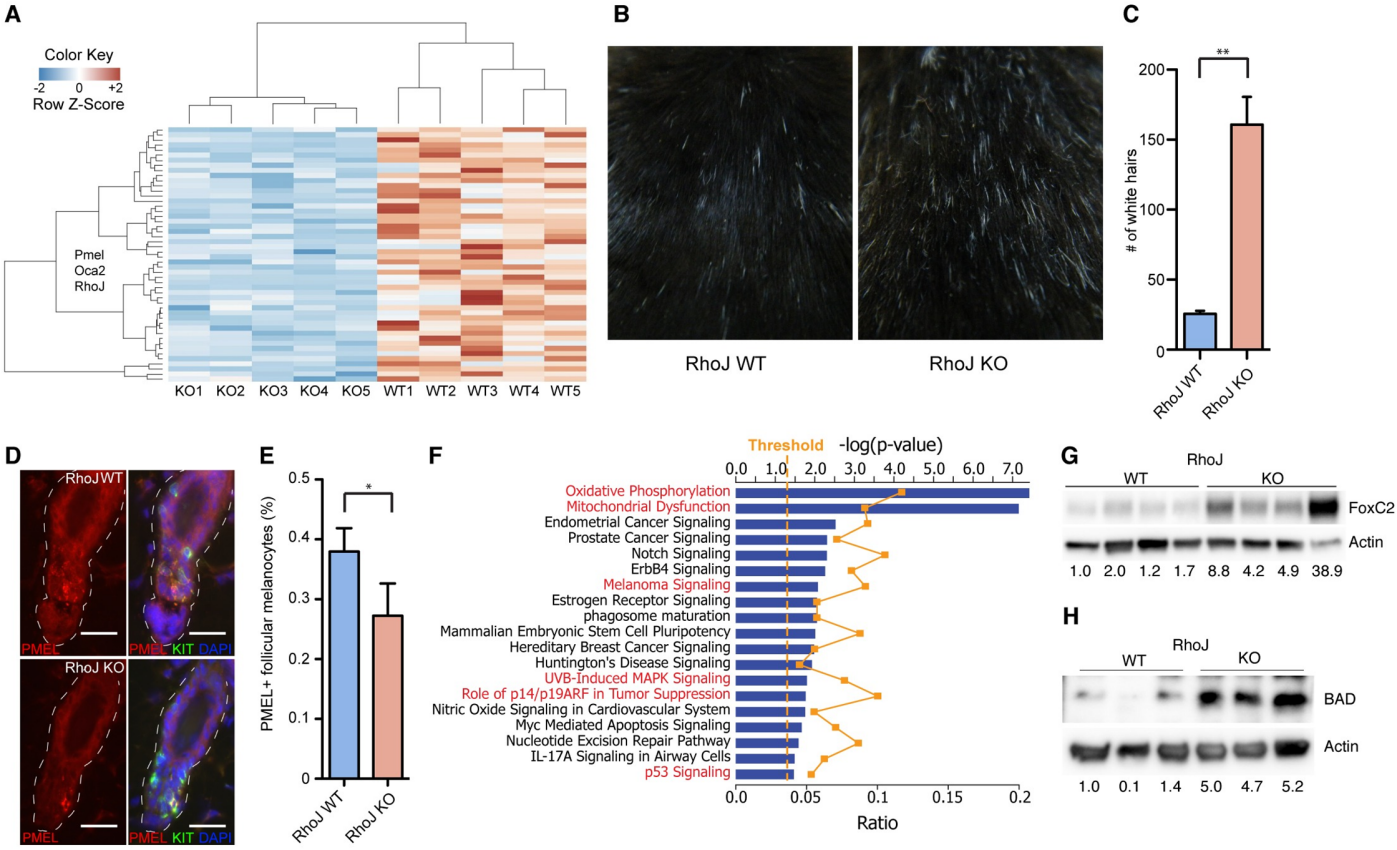
RhoJ modulates various signaling pathways to promote tumor growth. (A) Heat map plot of 50 modulated genes upon loss of RhoJ. Hierarchical clustering of RNA-seq normalized read counts ranging from less frequently expressed (dark blue) to overexpressed (dark red) genes. (B) RhoJ KO mice have a greater number of white hairs than RhoJ WT mice. Images of 8-month old mice show that loss of RhoJ induces accumulation of white hairs. (C) The number of white hairs over an area of 1 in^2^ were counted and quantified using ImageJ (**p = 0.002, Student’s t-test). Error bars indicate SEM. (D) Loss of RhoJ results in fewer PMEL17+ follicular melanocyte stem cells. Double labeling for the melanosome protein PMEL17 (red) in hair follicle melanocyte stem cells (KIT+, green) at telogen demonstrates a noticeable reduction in the intensity and extent of PMEL expression in RhoJ KO mice. White dashed line indicates the extent of the hair follicle. Scale bars: 10μm (E) Quantification of the immunolabeling described in D demonstrates a statistically significant difference between RhoJ WT and RhoJ KO hairs (*p = 0.03, Student’s t test). Error bars indicate standard deviation of the mean. (F) RhoJ regulates oxidative phosphorylation, melanocyte differentiation, and MAP kinase signaling. Based on the differentially expressed genes between melanoma tumors from RhoJWT and RhoJKO mice, Ingenuity Pathway Analysis (IPA) generated an enrichment of canonical pathways regulated by RhoJ based on the literature (pathways potentially regulated by BRAF are highlighted in red). Bars indicate the negative logarithm of the enrichment p-value. The orange dotted line indicated the statistical threshold (p<0.05) for the enrichment canonical pathways. The orange squares indicate the ratio (value of molecules in a given pathway that meet the cutoff criteria, divided by total number of molecules that make up that pathway) for each canonical pathway. (G) FoxC2 is up regulated in mouse tumor samples. Melanoma tumor lysates were prepared for western blot and probed for the indicated Abs. Relative densitometry values are shown below each blot. (H) BAD is up regulated in RhoJ KO mouse tumor samples. Melanoma tumor lysates were prepared for western blot and probed for the indicated Abs. Relative densitometry values are shown below each blot.

In order to better understand how RhoJ promotes tumor growth, the whole genome expression profiles of *Braf*^*V600E*^*;Pten*^*fl/+*^*;RhoJ*^*+/+*^ tumors and *Braf*^*V600E*^*;Pten*^*fl/+*^*;RhoJ*^*-/-*^ tumors were examined (Fig 2A, S2A Fig, complete list in S1 Dataset). Transcriptomics analysis revealed that RhoJ modulates the expression of pigment genes such as premelanosome protein (*Pmel17)* and Oca2 melanosomal transmembrane protein (*Oca2)* (Fig 2A). As a preliminary validation of the pathways identified in our transcriptomics experiments, we sought to determine whether RhoJ deletion affects melanogenesis. Initial observations revealed that RhoJ KO mice exhibit a greater number of white hairs as compared to RhoJ WT mice (Fig 2B and 2C, S2B Fig), indicating that RhoJ modulates either postnatal melanogenesis or melanocyte development. Immunofluorescence studies revealed that RhoJ KO mice contained fewer melanocytes that express Pmel17 (Fig 2D and 2E) yet have similar if not higher numbers of DCT-positive melanocyte stem cells within the hair follicle (S2C and S2D Fig) when compared to RhoJ WT mice. These results suggest that RhoJ modulates pigmentation by either causing a developmental melanocyte defect or by affecting melanocyte differentiation.

Interestingly, pathway enrichment analysis revealed that *RhoJ* deletion in mouse tumors modulated pathways that have previously been shown to be regulated by BRAF, such as cellular bioenergetics (oxidative phosphorylation) [23], RAF and PI3 kinase signaling pathways [24], UVB-induced MAPK signaling pathways [25], and p53 signaling pathways [20] (Fig 2F). Notably cytokines, immune regulators, and angiogenesis regulators were not identified in our bioinformatics analysis, suggesting that RhoJ regulates melanocyte growth in a cell autonomous manner. *FoxC2*, a gene that regulates the expression of multiple mitochondrial genes [26], was up-regulated in RhoJ KO tumors as compared to RhoJ wild type tumors as demonstrated by both RNA-seq results (Fig 2A) and by western blotting (Fig 2G). BRAF mutant cells are known to suppress AKT and BAD signaling to promote their growth *in vivo* [27]. Intriguingly, we observed that the expression of the proapoptotic protein BAD was elevated in RhoJ KO tumors (Fig 2H), consistent with the hypothesis that RhoJ downregulates BAD signaling. Similarly, other apoptosis genes were also modulated by RhoJ (see S1 Dataset). Taken together, these observations suggest that RhoJ modulates the growth of tumors by altering the metabolism and apoptotic threshold of BRAF mutant melanocytes (S2E Fig).

### RhoJ regulates the development of melanocytic nevi via a cell autonomous mechanism

**Fig 3:**
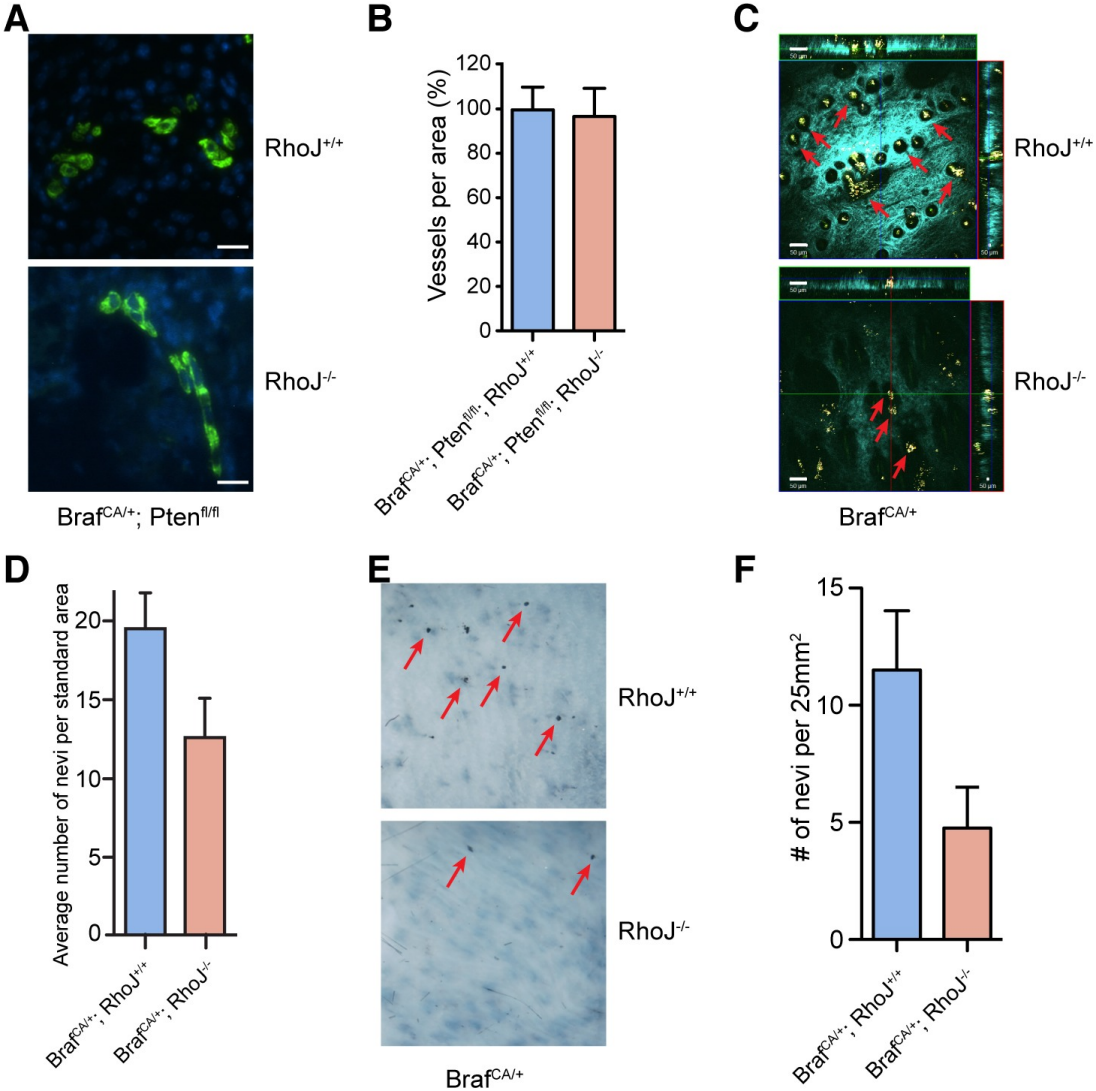
RhoJ regulates the formation of BRAF mutant nevi. (A) RhoJ deletion does not significantly affect the angiogenesis of BRAF mutant autcthonous melanoma tumors. Tumor sections from age matched (post natal day 30) BRAF^V600E^ and PTEN null RhoJ KO or RhoJ WT mice were stained with smooth muscle actin Ab followed by Alexa-488 secondary Ab to visualize blood vessels. Representative immunofluorescence sections are shown. Scale bars: 100μm. (B) RhoJ KO and RhoJ WT melanomas have similar numbers of vessels. The number of blood vessels in RhoJ WT and RhoJ KO per unit area (412μm x 412 μm) were quantified. The number of blood vessels were not significantly different (p = 0.8, n = 6 (mice) and 3 areas per tumor were counted). (C) RhoJ deletion inhibits nevus formation. MPM images were captured as described in materials and methods from *Tyr*:*Cre*^*ERT2*^*; Braf*^*V600E*^*; RhoJ*^*+/+*^ and *Tyr*:*Cre*^*ERT2*^*; Braf*^*V600E*^*; RhoJ*^*-/-*^. Colored lines indicate positions being displayed as xy (blue), xz (red) and yz (green) planes. Field of view is 636μm x 636μm Cyan: SHG of collagen; Green: fluorescence of keratin; Yellow and Red–fluorescence of melanin. Nevus indicated by red arrows. Scale bars: 50μm. (D) Nevi were quantified within the upper 50μm from 3-D skin reconstructions. Fewer microscopic nevi could be visualized from the skin surface in RhoJ KO skin as compared to RhoJ wild type skin (p = 0.059, ANOVA, n = 10). (E) RhoJ deletion reduced the number of macroscopic nevi visualized from the skin surface. Skin samples were fixed in 10% formalin for 36 hours and dehydrated in a series of increasing alcohol concentrations, and nevi were visualized by a dissecting microscope. Red arrows indicate pigmentation that could be visualized on the skin surface. (F) Macroscopic nevi were quantified on skin samples that measured 25mm^2^. RhoJ KO samples contained fewer nevi (p = 0.07, Student’s t-test, n = 4).

Our preliminary studies made the novel observation that RhoJ modulates melanocyte differentiation (Fig 2B). Previously published studies determined that RhoJ is expressed in endothelial cells in developing retinas [28], and RhoJ knockout mice exhibit delayed radial growth of the retinal vasculature without other discernible angiogenesis phenotypes [29]. Our transcriptomics analysis did not identify angiogenesis genes as a class that is modulated in RhoJ knockout tumors (Fig 2F), in contrast to previous studies that identified RhoJ as critical regulator of angiogenesis in xenograft models [22]. Analysis of the vasculature from RhoJ KO melanomas revealed no difference in the number of blood vessels per unit area in comparison to RhoJ WT melanomas (Fig 3A and 3B, S3A Fig), indicating that RhoJ deletion does not significantly affect tumor angiogenesis in our autochthonous mouse model. To further analyze the effects of RhoJ on melanocytes, RhoJ KO mice were crossed with a nevi mouse model that carries a *Tyr*:*Cre*^*ERT2*^ allele and a *Braf*^*CA*^ allele [18]. Nevi were quantified microscopically with multiphoton microscopy (MPM), a techniques that utilizes intrinsic fluorescent signals to generate a three-dimensional image of nevi [30], at the second telogen (p50) after the hair was removed. *Tyr*:*Cre*^*ERT2*^; *Braf*^*V600E*^*; RhoJ*^*-/-*^ mice had reduced the number of microscopic nevi that could be visualized from the skin surface as compared to *Tyr*:*Cre*^*ERT2*^; *Braf*^*V600E*^*; RhoJ*^*+/+*^ mice (Fig 3C and 3D, S3B Fig, S1 Video, S2 Video), a result that neared statistical significance (p = 0.059). Similarly, less nevi were visualized macroscopically in RhoJ knockout mice as compared to RhoJ wild type mice as visualized by a dissecting microscope (p = 0.07, Fig 3E and 3F, S3C Fig). Our observations indicate that although *RhoJ* is a global KO, its deletion has no detectable effect on the angiogenesis of autochthonous melanomas (S2C Fig). RhoJ deletion does affect the growth of melanoma tumors, the formation of nevi, and has subtle effects on melanocyte differentiation or development (S2D Fig). Taken together, these findings indicate that RhoJ specifically modulates the growth and survival of melanocytes.

### RhoJ is activated in a specific subset of melanoma tumors

**Fig 4:**
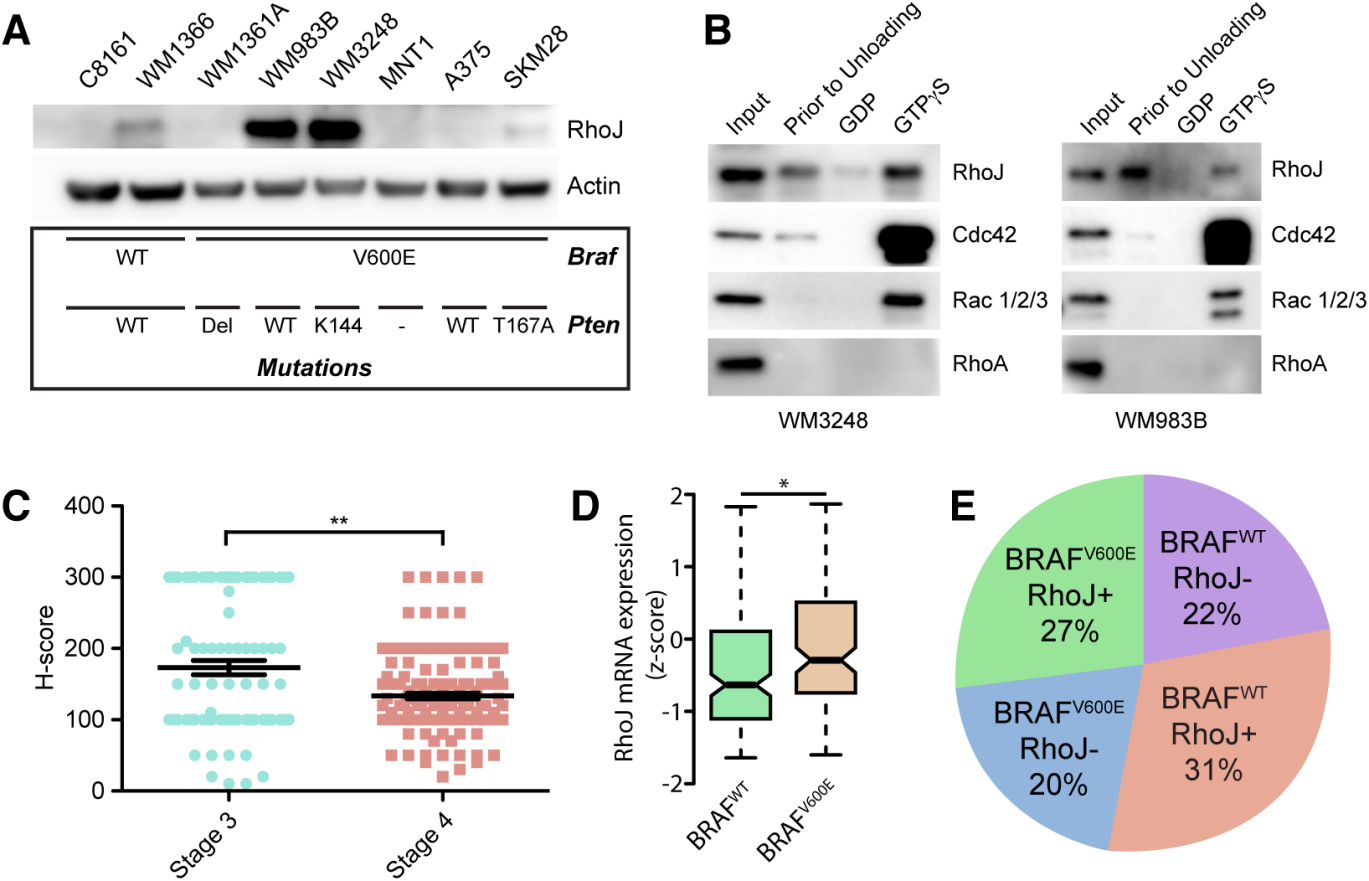
RhoJ signals through PAK1 and is expressed in a subpopulation of Braf mutant human tumors. (A) RhoJ is expressed in melanoma cell lines containing BRAFV^600E^ mts. Lysates from various melanoma cell lines containing WT BRAF or BRAFV600E mt were prepared and immunoblotted with the indicated Abs. BRAF and PTEN status are indicated below each cell line. (B) RhoJ is activated in melanoma cells. Melanoma cell lysates were analyzed in their initial state (“prior to unloading” lane) or after exchange and reloading with either GDP or GTPγS. Protein lysates were then incubated with PAK-PBD Agarose beads and precipitated. Precipitated lysates were subjected to immunoblotting using the indicated Abs. (C) RhoJ is expressed higher in stage III melanoma tumors. Tumors were scored for RhoJ expression and quantified using standard H-score criteria (**p<0.001). Stage IV tumors are a mixture of primary tumors and metastatic tumors. (D) RhoJ expression is higher in BRAF^V600E^ melanoma clinical specimens. TCGA SKCM RNA-seq data for RhoJ expression was compared between BRAF^WT^ (n = 168) and BRAF^V600E^ (n = 130) melanoma specimens (*p = 0.02, Student's t-test). (E) BRAF^V600E^ and RhoJ status among AJCC stage III and stage IV melanoma tumors. Tissue microarrays were stained with an anti-BRAF mutant specific anti-S100, and an anti-RhoJ Abs as indicated. The percentage of tumors that stained with S100 (positive control) Ab and also stained with RhoJ and/or the mutant BRAF Ab was calculated.

Although RhoJ is highly expressed in the heart and to a lesser extent in the liver [31], its expression in cutaneous melanoma has not been extensively analyzed. We examined the expression of RhoJ in a panel of human melanoma cell lines (Fig 4A) and determined that RhoJ expression levels varied widely in BRAF^V600E^ and BRAF^WT^ cells lines. Additional studies sought to determine whether RhoJ signaling was activated in melanoma cells that express high levels of RhoJ. Our previously published studies showed that RhoJ regulates PAK activity in human melanoma cell lines [20, 21]. Group I PAK kinases contain a p21-binding domain (PBD) where small GTPases, like Cdc42, RhoJ, and Rac, can bind when they are in their GTP-bound-active state [31]. We utilized a Cdc42-PAK functional assay to better evaluate the activation of this family of GTPases in melanoma and determined that RhoJ, Cdc42, and Rac1/2/3 interact with PAK in melanoma cells when they are GTP-bound but not when they are loaded with GDP (Fig 4B). Interestingly, we observed that RhoJ and Cdc42 bind to PAK conjugated agarose beads even when they are not loaded with GTP, suggesting that RhoJ and Cdc42 but not Rac1 are intrinsically activated in RhoJ highly expressing cells (Fig 4B, “prior to unloading” lane).

To verify that RhoJ plays a role in the growth of human melanomas, we optimized a RHOJ Ab for immunohistochemistry (S4A Fig) and examined the expression of RHOJ in AJCC stage III and stage IV melanoma tumors using two well-annotated melanoma tissue FFPE microarrays [32]. Stage III melanoma tumors expressed significantly higher levels of RHOJ than stage IV melanoma tumors (Fig 4C, S4B Fig). RhoJ is also expressed in some stage II lesions (S4C Fig), but large-scale examination of these lesions was limited by the number of that were available to test (these samples are often small in size and not included in most tissue microarrays). Integration of the Cancer Genome Atlas (TCGA) melanoma RNA-sequencing and exome-sequencing datasets confirmed a higher expression of RHOJ in BRAF^V600E^ as compared to BRAF^WT^ melanoma tissues (Fig 4D). Moreover, 27% of all tumors examined in a human melanoma tissue microarray had both the BRAF^V600E^ mutation and expressed high levels of RHOJ (Fig 4E). Taken together, these studies indicate that RHOJ is expressed in about half of stage III and stage IV melanoma tumors harboring BRAF^V600E^ mutation.

### PAK inhibitors selectively blocks the growth of RhoJ expressing melanomas by suppressing BAD phosphorylation

**Fig 5:**
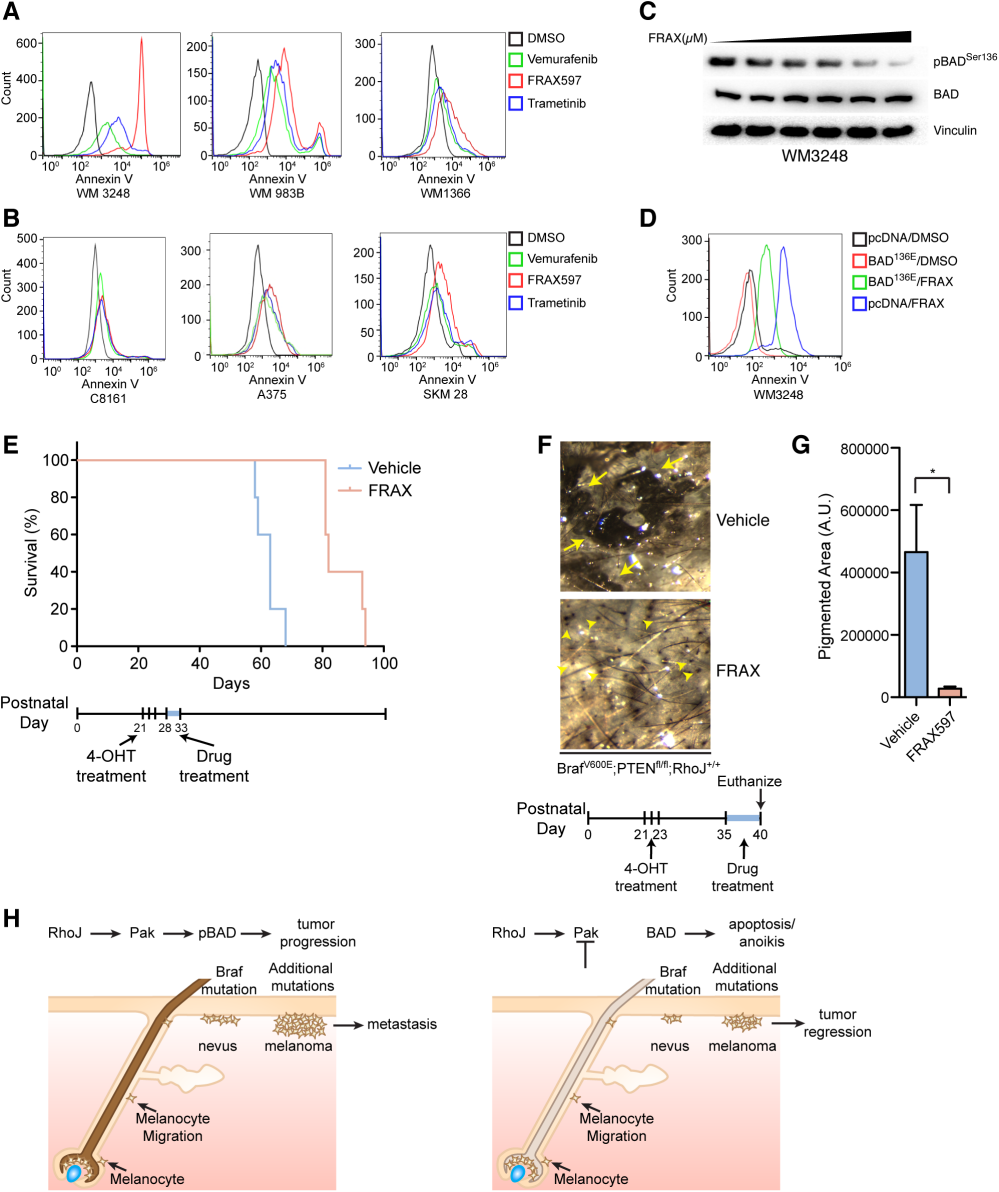
PAK inhibition induces apoptosis via BAD and blocks the progression of BRAF mutant melanomas. (A) FRAX597 inhibits PAK1 activation and induces apoptosis in BRAF^V600E^ melanoma cell lines. Apoptosis was quantified with annexin-V labeling and flow cytometry. (B) FRAX597 does not induce apoptosis in cell lines that do not express detectable RhoJ. Flow cytometry analysis of melanoma cell lines treated with vehicle, vemurafenib (5μM), FRAX597 (1μM), or trametinib (0.1μM). (C) PAK inhibition decreases the phosphorylation of BAD^Ser 136^. Melanoma cell line WM3248 harboring the BRAF^V600E^ mutation was treated with an increasing concentration of FRAX597 (0μM, 0.2μM, 0.5μM, 1μM, 2.5μM, 5μM) and immunoblotted with the indicated antibodies. (D) Over expression of pcDNA3 BAD^S136E^ phosphomimetic rescued survival after FRAX597 treatment. Cells were transfected with Lipofectamine 3000 according to the manufacturer’s instructions and treated with FRAX or DMSO. Apoptosis was measured with flow cytometry. (E) PAK inhibition prolongs survival in Braf^V600E^ PTEN null mice. Melanoma was induced in adult mice at P21, P22, and P23 with topical treatment of 4-OHT (25mg/mL) as previously described (18). Vehicle or FRAX597 (100mg/kg P.O. Q.D.) was administered for six days between P28 and P33. Mice were euthanized when the tumor burden exceeded the minimum restrictions as advised by veterinary technicians, and Kaplan-Meier survival curves were generated for *Braf*^*CA/+*^*; Pten*^*fl/fl*^*; Tyr*::*Cre*^*ERT2*^*; RhoJ*^*+/+*^ vehicle (n = 5) or FRAX597 (n = 5) treated animals. Log-rank test demonstrates significant difference between the two groups (**p<0.002, Log-rank test). (F) PAK inhibition delays tumor formation. Mice were treated with 4-OHT at P21, P22, and P23 on their backs to induce melanoma. Tumors were allowed to progress for 2 weeks prior to drug treatment. Mice were either administered FRAX597 or Vehicle for six days. Mice were euthanized after six days of drug treatment and images were taken with a dissecting microscope. Tumors were visible in vehicle treated mice (arrows) but smaller lesions were visible FRAX597 treated mice (arrowheads). (G) Disruption of RhoJ-PAK signaling blocks the formation of nevi and the growth of melanoma. The area of pigmented lesions (developing melanomas) from F was quantified (A.U. arbitrary units) and analyzed (*p = 0.04, n = 3, Student t-test). Error bars indicate SEM. (H) RhoJ expression enables melanocytes to differentiate and migrate out of the hair follicle during the process of nevogenesis and evolve into melanoma tumors. Perturbation of RhoJ-Pak signaling prevents melanocytes from differentiating normally, which leads to loss of pigment in hair, impairment of melanocytes to migrate out of the hair to form nevi, and prevent transformation into melanoma as efficiently.

Our initial studies demonstrated that RhoJ deletion slows the initiation and progression of BRAF mutant melanoma tumors *in vivo* (Figs 1 and 3). To determine whether blocking RhoJ signaling would be an effective method to inhibit the growth of melanoma tumors, we first examined whether blocking RhoJ signaling pharmacologically was sufficient to induce apoptosis *in vitro*. Melanoma cell lines were treated with a Group I selective PAK inhibitor, FRAX597 [33]. FRAX597 induced apoptosis in BRAF mutant cell lines that expressed high levels of RhoJ (WM3248 and WM983B), but not in BRAF wild type cell lines that expressed less RhoJ (WM1366, Fig 5A). Moreover, FRAX597 induced low levels of apoptosis in cells where RhoJ was undetectable, independently of the BRAF mutation status (Fig 5B). Previously published studies have defined doses of Vemurafenib and Trametenib that can induce apoptosis in a majority of treated cells *in vitro* after 72 hours [34]. Melanoma cell lines underwent apoptosis when they were treated with Vemurafenib for 72 hours (S5A Fig), consistent with work published by others [34]. FRAX597 induced more apoptosis than Vemurafenib and Trametenib after 24 hours in cell lines that expressed detectable levels of RhoJ (Fig 5A). Interestingly, increasing concentrations of FRAX597 inhibits the phosphorylation of ERK in BRAF mutant cell lines while it has less of an effect on an NRAS mutant cell line (S5B Fig), which suggests that RhoJ signaling may impact the MAP kinase pathway downstream of BRAF. In addition, we observed that PAK inhibition in NRAS mutant cell line WM1366 does induce apoptosis, but requires higher doses of drug (S5B Fig). Moreover, Pak inhibitors and BRAF inhibitors did not synergistically kill melanoma cells (S5C Fig), suggesting that the effect of PAK inhibitors on MAPK signaling may not be responsible for the observed apoptosis effect. PAK is known to inhibit apoptosis by inducing the phosphorylation of BAD at serine 136, which inhibits the ability of BAD to suppress the function of the anti-apoptotic protein BCL2 [35]. FRAX597 effectively inhibited the accumulation of BAD phosphorylated at Ser136 in a dose dependent manner (Fig 5C). Overexpression of a BAD^S136E^ phosphomimetic [36] protected cells from PAK-induced apoptosis (Fig 5D), indicating that PAK inhibitors induce apoptosis by blocking BAD phosphorylation.

To determine whether PAK inhibition could block the growth of early stage melanoma tumors, tumors were induced in adult mice as previously described [18] and treated with FRAX597 for six days either one week after tumors were induced or two weeks after tumor induction when tumors are first visible. Mice treated with FRAX597 for a short period of six days before tumors were visible significantly prolonged the survival of mice by 30 days (p<0.0021, Log-rank; Fig 5E), consistent with a role for RhoJ and PAK1 in the growth of nascent tumors. Treating mice with FRAX 597 for six days after tumors were first visible resulted in fewer tumors in FRAX597 treated mice as compared to vehicle treated mice (Fig 5F and S5D Fig) as measured by the pigmented area (Fig 5G). Taken together, these results identify PAK inhibitors as agents that can block the progression of early stage, BRAF mutant, RhoJ expressing melanomas by modulating BAD signaling.

## Discussion

Oncogene-induced senescence and apoptosis play an important role in inhibiting tumorigenesis by maintaining homeostasis [2]. In order to bypass homeostasis, tumors acquire mutations in oncogenes, such as BRAF, but also alter conventional signaling paradigms to facilitate growth. In this study, we sought to identify signaling pathways that allow melanoma cells to survive BRAF^V600E^ induced oncogenic stress. We identify a novel role for the Cdc42 family member RhoJ in resisting the effects of oncogene-induced stress. RhoJ deletion had mild effects on normal melanocyte differentiation and development, resulting in the accumulation of an increased number of grey hairs. RhoJ played an even more important role during the process of nevus formation and most significantly affected tumor development and metastasis. In this study, we were unable to examine whether RhoJ had an independent effect on tumor metastasis as we had no method to compare the number of metastasis in mice that had the same tumor burden. Future studies will address whether RhoJ has independent effects on tumor initiation and metastasis.

While previously published studies have defined a role for Cdc42 in regulating melanoma cell movement [37], this study represents the first identification of a specific Cdc42 signaling network that cooperates with BRAF to regulate melanocyte growth *in vivo*. The observation that RhoJ is not mutated in melanoma but yet plays a more important role in the growth of BRAF mutant cells ranging from nevi to tumors indicate that RhoJ is part of a normal signaling pathway that is co-opted to speed tumor development (Fig 5H). Interestingly, blocking RhoJ signaling had some effect on MAPK signaling, suggesting that RhoJ does influence the MAPK pathway. Future studies will examine how RhoJ cooperates with other MAPK drivers, such as NRAS, to promote tumor growth.

To better understand how RhoJ and PAK regulate tumor formation, we examined how RhoJ deletion affected gene expression in transformed melanocytes. Transcriptomic studies revealed that RhoJ knockout tumors had increased expression of genes involved in oxidative phosphorylation and the transcription factor FoxC2, which controls the expression of metabolic pathways. Deregulation of cellular energetics, a hallmark of cancer [38], has been postulated to be a mechanism to avoid oncogene-induced stress in melanoma [39]. Previous studies have also shown that biguanides such as metformin and phenformin, electron transport chain complex I inhibitors, when combined with BRAF inhibitors induce tumor regression in BRAF^V600E^/PTEN^fl/fl^ mouse model of melanoma [40]. The observation that RhoJ modulates the expression of complex I components (S2E Fig) but does not affect tumor angiogenesis in our model (Fig 3A) identifies RhoJ signaling as a novel regulator of metabolic adaptation in BRAF mutant melanoma cells.

In addition to modulating the expression of oxidative phosphorylation genes, loss of RhoJ also led to an upregulation of the pro-apoptotic gene BAD. PAK is known to activate BAD phosphorylation, promoting BAD binding to 14-3-3τ instead of Bcl2, resulting in increased survival [35]. Overexpression of a BAD phosphomimetic mutant protected cells from the effects of a PAK inhibitor (Fig 5E), verifying that PAK inhibitors modulate cell survival by regulating BAD activity. We observed that RhoJ knockout tumors induced BAD expression, while acute inhibition of PAK modulated BAD activity but not BAD levels. These results are likely a consequence of chronic versus acute inhibition of RhoJ and PAK signaling, respectively. Anoikis occurs when cells can no longer adhere to the extracellular matrix and undergo apoptosis [41]. Migrating cancer cells leave the primary niche and avoid anoikis because they express mutated oncogenes and other genes that block apoptosis induction [42]. Previous studies have demonstrated that the BRAF oncogene protects melanoma cells from anoikis by modulating BAD signaling [43]. Similarly, PAK activation, which is overexpressed in breast cancer, is known to protect MCF10A cells from undergoing anoikis [44]. Taken together, our studies identify RhoJ and PAK as critical regulators of both metabolic adaptation and apoptosis evasion, key events required to establish the tumorigenic platform.

To validate whether the RhoJ-PAK signaling network influenced the growth of BRAF mutant tumors, we determined whether PAK inhibitors could block the growth of BRAF mutant autochthonous mouse melanomas during early phases of development. The PAK inhibitor FRAX597 inhibits the growth of nascent tumors *in vivo* after only six days of treatment (Fig 5E) and induces apoptosis *in vitro* after 24 hours (Fig 5A) by blocking BAD phosphorylation (Fig 5C). While FRAX597 does have some effects on modulating MAP kinase signaling, these agents do not synergize with Vemurafenib to kill melanoma cells suggesting that these agents induce apoptosis by modulating BAD and not by modulating MAPK signaling. Interestingly, FRAX597 has more dramatic effects on MAPK signaling in BRAF mutant cells as compared to NRAS mutant cells, suggesting that these agents may also impact signaling downstream of BRAF (S5B Fig). Future studies will examine the connections between RhoJ-PAK signaling and MAPK signaling. Finally, administering FRAX597 after tumors were visible also affected tumor growth (Fig 5G), suggesting that PAK and RhoJ modulate both the phases of tumor initiation and tumor progression.

It is interesting to note that the results presented here are more dramatic than other studies which demonstrated that the BRAF inhibitor PLX4720, the precursor derivative of Vemurafenib, only moderately inhibited tumor growth *in vivo* without inducing tumor regression [45] or preventing relapse [27]. These results again suggest that FRAX597 inhibits tumor growth by a mechanism other than altering MAPK signaling. Of note, while the MEK inhibitor PD325901 can induce tumor regression and prolong survival in transgenic mouse models, this requires long-term treatment (six weeks) [18]. Previous studies have documented that PAK1 is amplified in BRAF wild type tumors [46] and identified PAK inhibitors as agents that can slow the growth of melanoma xenografts [47] and synergize with BRAF inhibitors to kill melanoma cells [48]. In contrast, the studies presented here define a role for RhoJ signaling in regulating the growth of BRAF mutant melanocytes at all stages (Fig 5H), and result in the remarkable observation that early, limited treatment with PAK inhibitors is effective in halting tumor growth. Our tissue microarray studies determine that RhoJ is more highly expressed in stage III disease as compared to stage IV disease, suggesting that RhoJ is likely a better therapeutic target in early stage melanomas. Future studies will involve assembling tissue collections of early stage melanomas to verify whether RhoJ is an ideal therapeutic target for early stage disease and determine whether RhoJ inhibition blocks both tumor growth and metastasis.

## Materials and methods

### Mice

RhoJ KO mice were generated as previously described [29]. Briefly, the ES cells used to generate RhoJ knockout mice were derived from C57Bl/6N-*RhoJ*^*tm1a(KOMP)Wtsi*^ and injected into C57B6/J mice to generate chimeric mice. The melanoma prone mice have the genetic background C57BL/6.Cg- *Braf*^*tm1Mmcm*^ *Pten*^*tm1Hwu*^ *Tg(Tyr-cre/ERT2)13Bos/BosJ* (*Braf*^*CA*^, *Pten*^*lox4-5*^, *Tyr*::*Cre*^*ERT2*^) that have been previously characterized [18]. Both mice strains were crossed to generate *Braf*^*CA*^, *Pten*^*lox4-5*^, *Tyr*::*Cre*^*ERT2*^, *RhoJ*^*-/-*^ mice. The entire survival study utilized a total of 19 males and 37 females. The pigmentation of the mouse paws were analyzed by measuring the area of the pigmented region in the paws and normalizing to the entire paw using ImageJ software. The quantification of the paws includes all the paws shown in the figures and supporting figures.

### Multiphoton microscopy of mouse skin

*Tyr*:*Cre*^*ERT2*^; *Braf*^*V600E*^*; RhoJ*^*+/+*^ and *Tyr*:*Cre*^*ERT2*^; *Braf*^*V600E*^*; RhoJ*^*-/-*^ mice were shave depilated at p50 (second telogen), euthanized, and immediately imaged ex-vivo (no labeling) with MPM to capture the fluorescence signal from keratin and melanin and second harmonic generated (SHG) signal from collagen using LSM 510 NLO Zeiss system. Fluorescence and second harmonic generation was excited by femtosecond Titanium: Sapphire (Chameleon-Ultra, Coherent) laser at 900 nm. Emission was detected at 390–465 nm for SHG channel (blue), and 500–550 nm (green) and 565–650 (red) fluorescence channels. Each animal was imaged at 8 to 10 randomly chosen locations on depilated skin of the lower back. Stacks of optical sections of 636μm x 636μm at different depths ranging from 0 to 240 μm (5 μm steps) were obtained to allow for 3-D volume reconstruction (LSM Image Browser, Carl Zeiss GMBH).

### Activation of Tyr::Cre^ERT2^ transgene and treatment of mice with PAK inhibitors

*Braf*^*CA*^, *Pten*^*lox4-5*^, *Tyr*::*Cre*^*ERT2*^ mice were genotyped as previously described [18]. The Cre-specific primers are: forward 5’- GGTGTCCAATTTACTGACCGTACA-3’ and reverse 5’- CGGATCCGCCGCATAACCAGTG -3’. Topical administration of 4-hydroxytamoxifen (4-OHT) to the back skin on pups and adults was performed as previously described [18]. Paws of mice were also treated with 4-OHT at P2, P3, and P4. FRAX597 was administered to mice daily via oral gavage daily at a dose of 100 mg/kg [33] and prepared in 60%:40% PEG-400:DI water. Tumors in adult mice (P21) were induced for three days and allowed to progress for one week, at which point mice began FRAX treatment for six days. The veterinary staff was blinded as to the genotype of experimental animals utilized in the study, and monitored experimental animals on a daily basis. Animals were culled when tumor burden reached ethical limits and if the animals displayed signs of ill health or distress as determined by the veterinary staff. All mouse procedures were approved by UCI’s IACUC regulations and standards. As an additional control for background strain variability, all survival comparisons were made between littermates.

### RNA sequencing

Total RNA from RhoJ WT 1–5 and RhoJ KO 1–5 was monitored for quality control using the Agilent Bioanalyzer Nano RNA chip (Santa Clara, CA). Samples with a RIN number >8 were used to construct libraries using the Illumina TruSeq RNA v2 kit. The input quantity for total RNA was 1μg and mRNA was enriched using oligo dT magnetic beads. The enriched mRNA was chemically fragmented for four minutes. First strand synthesis used random primers and reverse transcriptase to make cDNA. After second strand synthesis the ds cDNA was cleaned using AMPure XP beads (Beckman Coulter, Beverly, MA) and the cDNA was end repaired and then the 3’ ends were adenylated. Illumina barcoded adapters were ligated on the ends and the adapter-ligated fragments were enriched by nine cycles of PCR. The resulting libraries were validated by qPCR and sized by Agilent Bioanalyzer DNA high sensitivity chip. The concentrations for the libraries were normalized and then multiplexed together. The concentration for clustering on the flow cell was 12.5pM. The multiplexed libraries were sequenced on three lanes using paired end 100 cycles chemistry for the Illumina HiSeq 2500. The version of HiSeq control software was HCS 2.2.38 with real time analysis software, RTA 1.18.61.

### Immunohistochemistry, immunofluorescence and immunoblotting

RhoJ staining was optimized at 1:1000 using an Ab from Genetex (Irvine, CA). Slides were developed with either a horse-radish peroxidase liquid 3,3'-diaminobenzidine chromogen system (DAKO, Carpirteria, CA) or an alkaline phosphatase liquid permanent red chromogen system (DAKO), according to the manufacture’s protocol. Fluorescence labeling was performed using SMA (Abcam 1:100) PMEL, KIT, DCT, and DAPI as described [49]. The expression or phosphorylation of proteins was detected by western blotting using the following Abs: RhoJ (Abnova 1:250), Cdc42, Rac1/2/3, RhoA, actin, BAD, phospho-Bad^Ser138^, and Vinculin (all from cell signaling 1:1000).

### Cdc42 activity assays

A Cdc42 activation assay was performed according the manufacturer’s protocol (Cell Biolabs, San Diego, CA). Briefly, cell lysates expressing high RhoJ were unloaded of guanosine nucleotides and either loaded with GDP or GTPγS. Agarose beads conjugated with the PAK1 PBD domain pulled down Cdc42, Rac, or RhoJ only when GTP-bound. Lysates were then immunoblotted with indicated Abs.

### Flow cytometric analyses for apoptosis

Approximately 1 × 10^6^ melanoma cell treated with FRAX-597 were trypsinized and washed with PBS, the single-cell suspensions were incubated with Alexa Fluor-Annexin V and propidium iodide (PI) (V13245; Invitrogen) per the manufacturer’s protocol and were subjected to flow cytometric analysis. In all cases, cell debris was gated out based on forward scatter and side scatter analysis. Data was analyzed using FlowJo (Ashland, OR). Cells not treated with FRAX-597 were used to establish gating parameters. WM3248 cells were transfected with BAD^S136E^ (3582 pcDNA3 BAD^S136E^ Addgene #8800), with Lipofectamine 3000 (Invitrogen) according to the manufacturer’s protocol. On the day of transfection, 14μg of DNA from the respective constructs were incubated with Plus Reagent in Opti-MEM to a volume of 350μL, and then mixed with 43μL lipofectamine in 350μL Opti-MEM and incubated for 20 min at room temperature. The DNA-Lipofectamine complexes were then added to each plate, and the cells were incubated at 37°C in a CO_2_ incubator. The media was replaced after 24hrs and cells selected in G418.

### Statistical analysis

The power and sample size calculations for the survival studies (Fig 1A and 1B) were based on a simulation of 1000 datasets utilizing SAS v9.2. To calculate the power, the simulation under the alternative hypothesis made the following assumptions: 1.) No censoring data for both groups; 2.) The first event occurred on day 30 for the controls; 3.) The first event occurred on day 40 for RhoJ^-/-^; 4.) Hazard ratios used were 1.5, 2, 4, 6; and 5.) Sample size of mice per group was 15, 20, 25, 30, 40, and 50. To calculate the type I error (alpha) under the null hypothesis the following assumptions were made: 1.) Data was not censored for both groups; 2.) The first event occurred on day 30 for both groups; 3.) Hazard ratio is 1; and 4.) Sample size of the mice per group was 15, 20, 25, 30, 40, and 50.

Continuous outcomes were summarized via mean and standard error and tests of unadjusted means between groups were conducted using a two-sample t-test with unequal variances. Mean RhoJ expression levels adjusting for stage, age and gender were estimated and compared using multiple linear regression analysis. Residual diagnostics were performed in order to assess the functional form of continuous covariates included in the model and to identify potentially influential subjects. Estimated mean differences, corresponding Wald-based 95% confidence intervals, and p-values corresponding to the test of no association in RhoJ levels were reported for all model covariates. No deviations in model fit or influential observations were observed.

The TCGA analysis was performed as follows. The skin cutaneous melanoma (SKCM) TCGA Exome-sequencing and RNA-sequencing datasets were downloaded June 2016. Single nucleotide variants (SNVs) at the position 1799 of the BRAF gene (c.1799T>C) leading to a non-synonymous alteration at the amino acid 600 of the BRAF protein were annotated as BRAFV600E. Additional, RhoJ expression assessed by RNA-sequencing was normalized using the z-score and the statistical difference on mean expression between BRAF wt and BRAFV600E was assessed by the Student's t-test.

### Study approval

Mice were housed and maintained by the University Laboratory Animal Resources. All animal experiments were approved by the University of California, Irvine’s Institutional Animal Care and Use Committee. Melanoma tumor microarray tissues were collected and processed with approval from the Providence Saint John’s Health Center and John Wayne Cancer Institute joint Institutional Review Board and Western Institutional Review Board.

## Supporting information

S1 Fig RhoJ regulates melanoma tumor development. (A) Inducible melanoma mouse model containing Braf mutation (mt), loss of Pten and RhoJ. Mice carrying *Braf*^*CA/+*^, *Pten*^*fl/fl*^ and *Tyr*:*Cre*^*ERT2*^ alleles were crossed with constitutive RhoJ KO mice. Activation of Cre^ERT2^ by 4-OHT leads to a Braf^V600E^ mutation and Pten loss. (B) RhoJ is not expressed in Rhoj KO mice. Formalin-fixed paraffin embedded mouse melanoma skin was bleached with 3% hydrogen peroxide (overnight) to remove melanin and analyzed for expression of RhoJ. (C) RhoJ KO mice have reduced number of lung metastases. Representative lungs from aged-matched mice (P30) were stained with H&E in *Braf*^*CA/+*^*; Pten*^*fl/fl*^*;Tyr*::*CreER; RhoJ*^*+/+*^ (top panels) and *Braf*^*CA/+*^*; Pten*^*fl/fl*^*;Tyr*::*CreER; RhoJ*^*-/-*^ (bottom panels) animals. Melanoma cells were identified by their pigmentation characteristic. Scale bar is 200μm. (D) RhoJ deletion delays melanoma development. Paws of 4-OHT treated mice were imaged at 10, 17, and 24 days post birth. The mice depicted were utilized to generate the graphs shown in Fig 1D. (E) RhoJ KO paws eventually reach the same level of pigmentation as RhoJ WT paws. Paws of 4-OHT treated RhoJ KO mice were imaged at P33.
(TIF)

S2 Fig RhoJ modulates various signaling pathways to promote tumor growth. (A) Heat map of 50 different modulated genes upon loss of RhoJ. Hierarchical clustering of RNA-seq count reads ranging from less frequently expressed (dark blue) to overexpressed (dark red). (B) RhoJ KO mice have a greater number of white hairs than RhoJ WT mice. Images of 8-month old mice show that loss of RhoJ induces accumulation of white hairs. (C) Melanocyte stem cells reside in the hair germ of RhoJ KO hair follicles. Both RhoJ WT and RhoJ KO skins exhibit telogen stage hair follicles that contain DCT+ (red) McSCs. White dashed line indicates the extent of the hair follicle. Scale bars: 10μm. (D) Quantitative analysis of DCT+ cells that reside in the hair follicle or RhoJ KO and RhoJ WT mice are represented as a box and whisker box plot. The box plot is the 25-75^th^ percentile and the whiskers are the min and the max. (E) Bioenergetic genes, found throughout the electron transport chain, upregulated when RhoJ is absent are shown. Note the number of genes that are present in complex I.
(TIF)

S3 Fig RhoJ has a cell autonomous effect on melanocytes independent of angiogenesis. (A) RhoJ expression does not affect the number blood vessels. Tumor sections from age matched (post natal day 30) BRAF^V600E^ and PTEN null mice were stained with smooth muscle actin followed by Alexa-488 secondary antibody to visualize blood vessels. All stained sections are shown (Field of view 412μm x412μm). Scale bars: 100μm. (B) RhoJ deletion inhibits nevus formation. MPM images were captured as described in materials and methods from BRAF^V600E^; RhoJ^-/-^ mouse skin. Colored lines indicate positions being displayed as xy (blue), xz (red) and yz (green) planes. Field of view is 636μm x 636μm Cyan: SHG of collagen; Green: fluorescence of keratin; Yellow and Red–fluorescence of melanin. Nevus indicated by red arrows. Scale bars: 50μm. (C) RhoJ deletion reduced the number of nevi that could be visualized on the skin surface. Skin samples were fixed in 10% formalin for 36 hours and dehydrated in a series of increasing alcohol concentrations and imaged using a dissecting microscope to visualize nevi on the skin surface. Red arrows indicate a nevus.
(TIF)

S4 Fig RhoJ is expressed in a subpopulation of Braf mutant human tumors. (A) Optimization of RhoJ antibody for immunohistochemistry evaluation of AJCC stage III and IV TMAs. Human melanoma tumors were stained with an optimized RhoJ Ab and developed with liquid permanent red. Representative samples with the indicated H-score were determined by a dermatopathologist. (B) Over 50% of human melanomas express RhoJ. Quantification of RhoJ+ tumors were based on H-score. (C) Stage II melanomas express RhoJ. Stage II TMA were obtained from US Biomax (ME481a) and developed with DAB.
(TIF)

S5 Fig Pak inhibition induces apoptosis via BAD and blocks the progression of BRAF mutant melanomas. (A) Melanoma cells undergo apoptosis with a 72 hour treatment of FRAX597, Vemurafenib, or Trametinib. All of the cells from WM3248 underwent apoptosis when treated with FRAX597 by 72 hours and is not shown in the graph. (B) FRAX597 inhibits Pak1 activation and induces apoptosis in BRAF^V600E^ melanoma cell lines. Melanoma cell lines harboring either BRAF^V600E^ or BRAF^WT^ were treated with increasing concentrations of FRAX597 (0μM, 0.2 μM, 0.5 μM, 1 μM, 2.5 μM, 5 μM) and immunoblotted with the indicated Abs to measure Pak1 activation (pMEK^Ser298^) and apoptosis (cleaved PARP). (C) FRAX597 does not synergize with Vemurafenib. Cells were treated with either FRAX597, Vemurafenib, or both and processed with FACS. (D) Pak inhibition delays tumor formation. Melanoma was induced as described Fig 5F and administered with vehicle or FRAX597 via oral gavage. Skin images were captured using a dissection scope.
(TIF)

S1 Dataset List of genes regulated by RhoJ based on RNA-sequencing analysis. RNA samples were obtained from RhoJ wildtype (n = 5) and knockout (n = 5) mice and subjected to RNA sequencing and analysis with Tuxedo Suite as previously described [50]. Briefly, the RNA-sequencing reads were aligned to the mouse (Mus musculus; mm10) genome using TopHat. Transcripts were assembled and compared with Cufflinks and Cuffdiff, respectively, to find differentially expressed genes between the two groups. Apoptosis tab: List of apoptosis genes regulated by RhoJ.
(XLSX)

S1 Video RhoJ regulates the formation of BRAF mutant nevi. Stacks of optical sections of 636μmx636μm at different depths ranging from 0 to 240 μm (5 μm steps) were obtained to allow for 3-D volume reconstruction (LSM Image Browser, Carl Zeiss GMBH). A representative 3-D volume reconstructed movie of *Tyr*:*Cre*^*ERT2*^*; Braf*^*V600E*^*; RhoJ*^*+/+*^ is shown. Cyan: SHG of collagen; Green: fluorescence of keratin; Yellow and Red–fluorescence of melanin.
(AVI)

S2 Video RhoJ regulates the formation of BRAF mutant nevi. Stacks of optical sections of 597 636μmx636μm at different depths ranging from 0 to 240 μm (5 μm steps) were obtained to 598 allow for 3-D volume reconstruction (LSM Image Browser, Carl Zeiss GMBH). A representative 3-D volume reconstructed movie of *Tyr*:*Cre*^*ERT2*^*; Braf*^*V600E*^*; RhoJ*^*-/-*^ is shown. Cyan: SHG of collagen; Green: fluorescence of keratin; Yellow and Red–fluorescence of melanin.
(AVI)
