# Supplementary figures and images for: The RhoJ-BAD signaling network: An Achilles’ heel for BRAF mutant melanomas
Source: PLoS Genet. 2017 Jul 28;13(7):e1006913. doi: 10.1371/journal.pgen.1006913 (PMC5549996; doi:10.1371/journal.pgen.1006913)

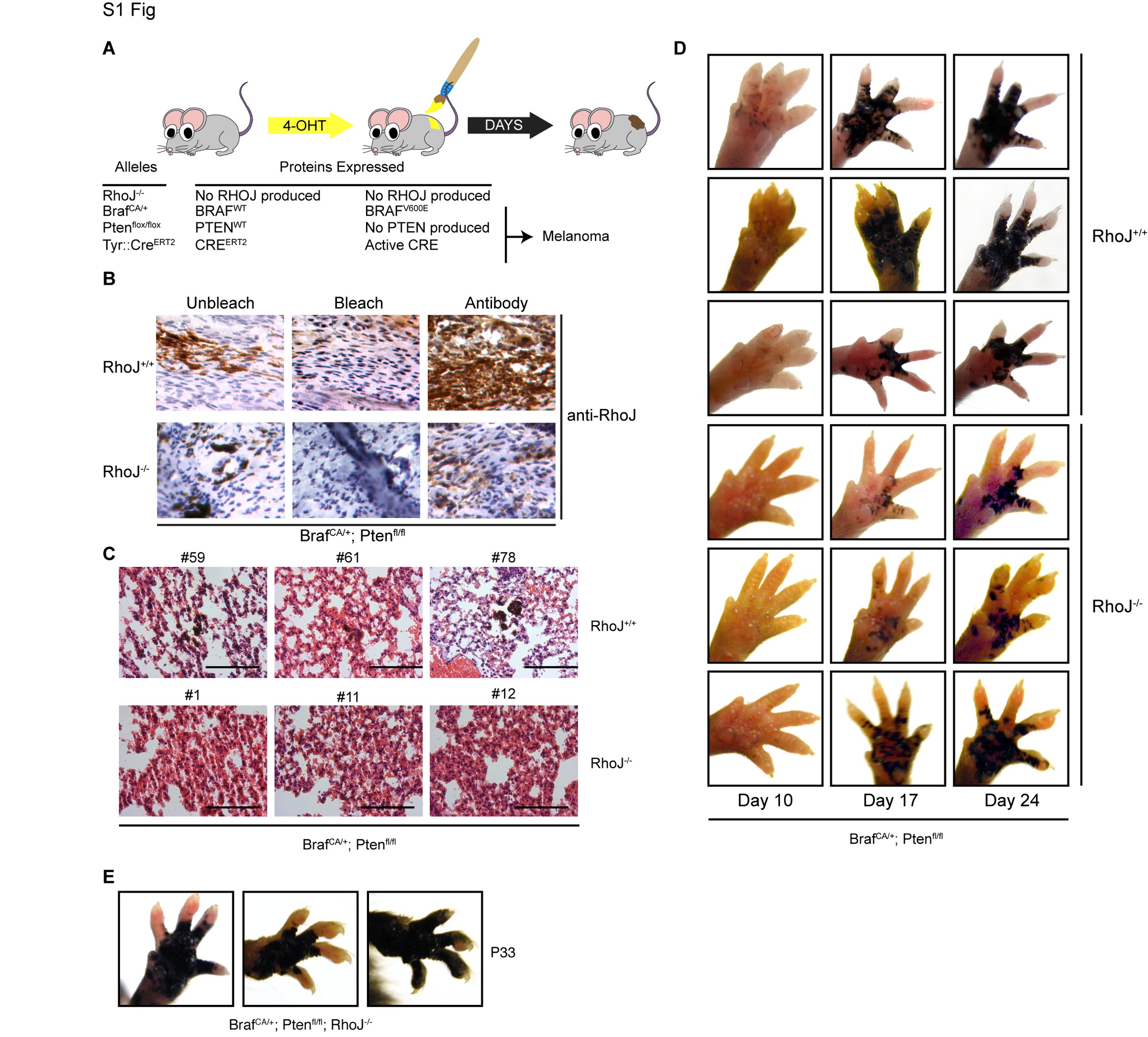

Supplement: S1 Fig — (A) Inducible melanoma mouse model containing Braf mutation (mt), loss of Pten and RhoJ. Mice carrying BrafCA/+, Ptenfl/fl and Tyr:CreERT2 alleles were crossed with constitutive RhoJ KO mice. Activation of CreERT2 by 4-OHT leads to a BrafV600E mutation and Pten loss. (B) RhoJ is not expressed in Rhoj KO mice. Formalin-fixed paraffin embedded mouse melanoma skin was bleached with 3% hydrogen peroxide (overnight) to remove melanin and analyzed for expression of RhoJ. (C) RhoJ KO mice have reduced number of lung metastases. Representative lungs from aged-matched mice (P30) were stained with H&E in BrafCA/+; Ptenfl/fl;Tyr::CreER; RhoJ+/+ (top panels) and BrafCA/+; Ptenfl/fl;Tyr::CreER; RhoJ-/- (bottom panels) animals. Melanoma cells were identified by their pigmentation characteristic. Scale bar is 200μm. (D) RhoJ deletion delays melanoma development. Paws of 4-OHT treated mice were imaged at 10, 17, and 24 days post birth. The mice depicted were utilized to generate the graphs shown in Fig 1D. (E) RhoJ KO paws eventually reach the same level of pigmentation as RhoJ WT paws. Paws of 4-OHT treated RhoJ KO mice were imaged at P33. (TIF) [file pgen.1006913.s001.tif]

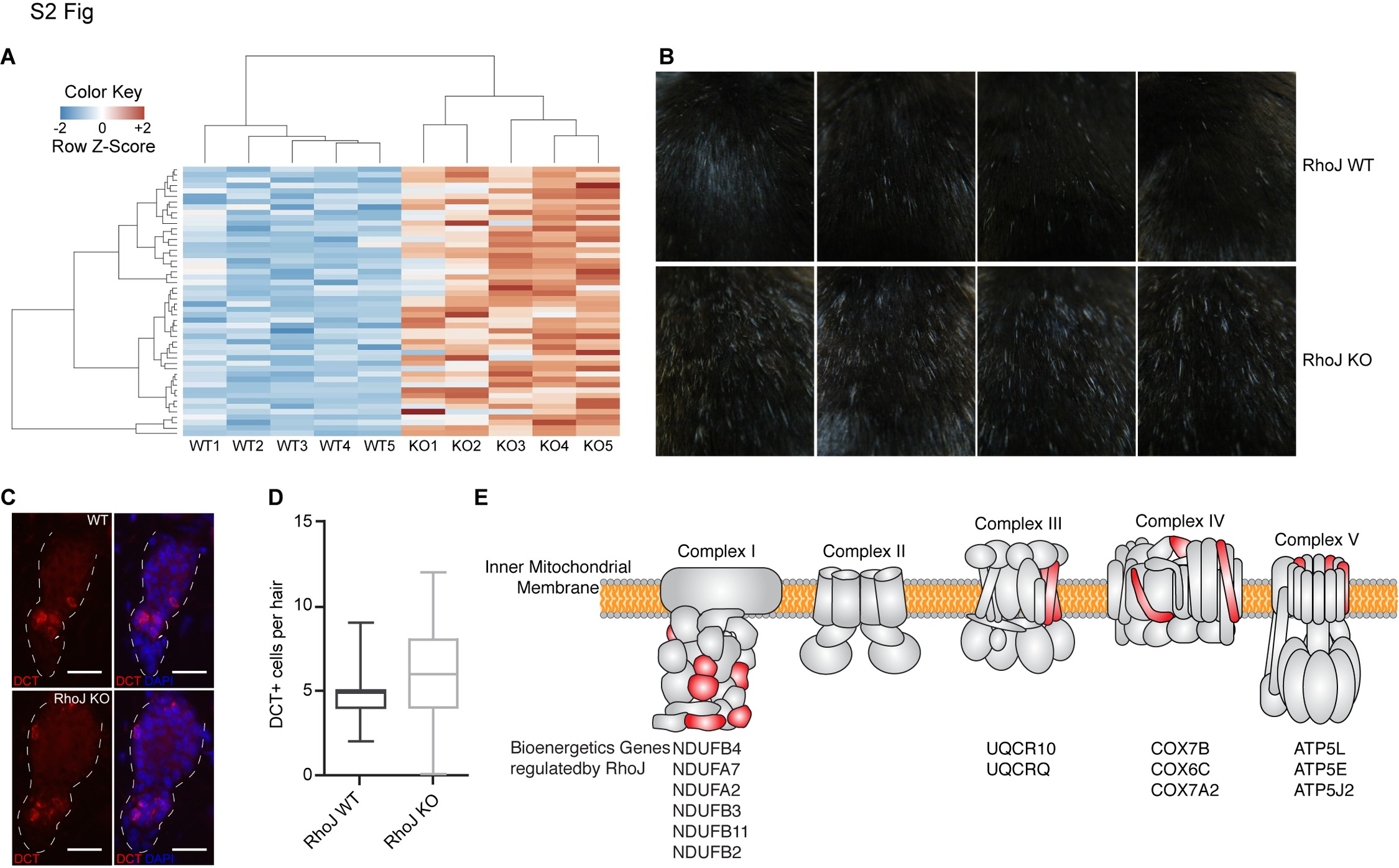

Supplement: S2 Fig — (A) Heat map of 50 different modulated genes upon loss of RhoJ. Hierarchical clustering of RNA-seq count reads ranging from less frequently expressed (dark blue) to overexpressed (dark red). (B) RhoJ KO mice have a greater number of white hairs than RhoJ WT mice. Images of 8-month old mice show that loss of RhoJ induces accumulation of white hairs. (C) Melanocyte stem cells reside in the hair germ of RhoJ KO hair follicles. Both RhoJ WT and RhoJ KO skins exhibit telogen stage hair follicles that contain DCT+ (red) McSCs. White dashed line indicates the extent of the hair follicle. Scale bars: 10μm. (D) Quantitative analysis of DCT+ cells that reside in the hair follicle or RhoJ KO and RhoJ WT mice are represented as a box and whisker box plot. The box plot is the 25-75th percentile and the whiskers are the min and the max. (E) Bioenergetic genes, found throughout the electron transport chain, upregulated when RhoJ is absent are shown. Note the number of genes that are present in complex I. (TIF) [file pgen.1006913.s002.tif]

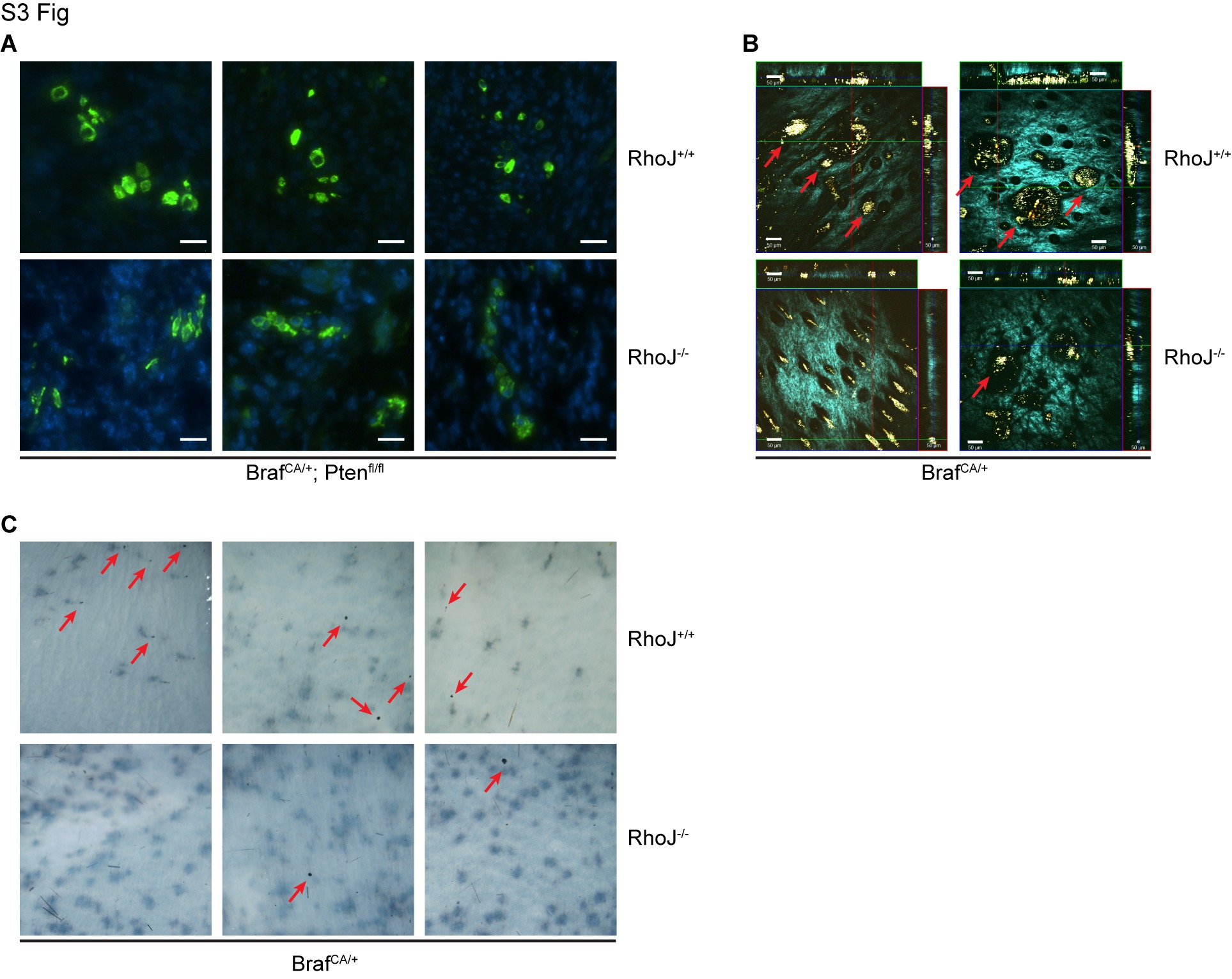

Supplement: S3 Fig — (A) RhoJ expression does not affect the number blood vessels. Tumor sections from age matched (post natal day 30) BRAFV600E and PTEN null mice were stained with smooth muscle actin followed by Alexa-488 secondary antibody to visualize blood vessels. All stained sections are shown (Field of view 412μm x412μm). Scale bars: 100μm. (B) RhoJ deletion inhibits nevus formation. MPM images were captured as described in materials and methods from BRAFV600E; RhoJ-/- mouse skin. Colored lines indicate positions being displayed as xy (blue), xz (red) and yz (green) planes. Field of view is 636μm x 636μm Cyan: SHG of collagen; Green: fluorescence of keratin; Yellow and Red–fluorescence of melanin. Nevus indicated by red arrows. Scale bars: 50μm. (C) RhoJ deletion reduced the number of nevi that could be visualized on the skin surface. Skin samples were fixed in 10% formalin for 36 hours and dehydrated in a series of increasing alcohol concentrations and imaged using a dissecting microscope to visualize nevi on the skin surface. Red arrows indicate a nevus. (TIF) [file pgen.1006913.s003.tif]

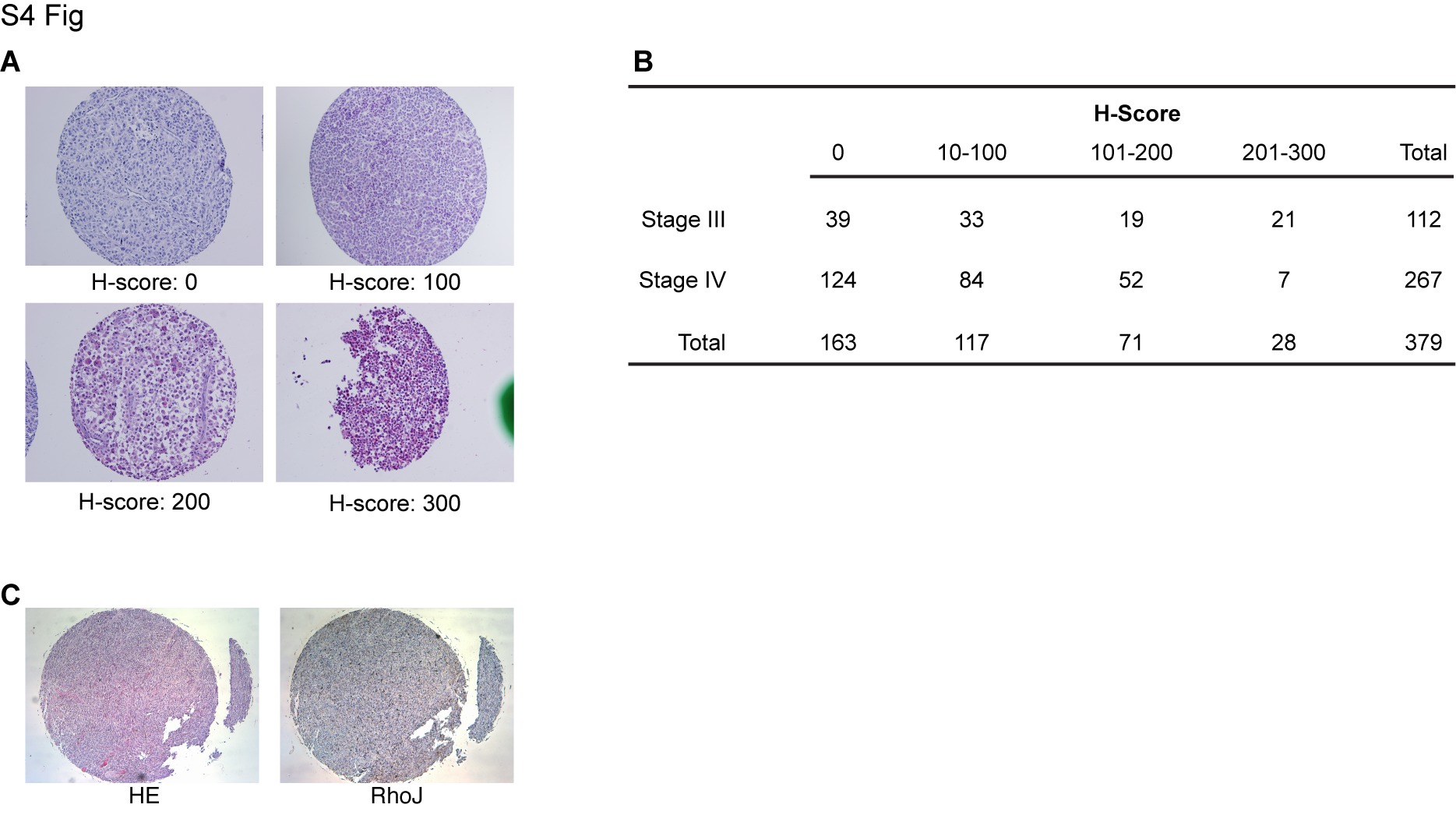

Supplement: S4 Fig — (A) Optimization of RhoJ antibody for immunohistochemistry evaluation of AJCC stage III and IV TMAs. Human melanoma tumors were stained with an optimized RhoJ Ab and developed with liquid permanent red. Representative samples with the indicated H-score were determined by a dermatopathologist. (B) Over 50% of human melanomas express RhoJ. Quantification of RhoJ+ tumors were based on H-score. (C) Stage II melanomas express RhoJ. Stage II TMA were obtained from US Biomax (ME481a) and developed with DAB. (TIF) [file pgen.1006913.s004.tif]

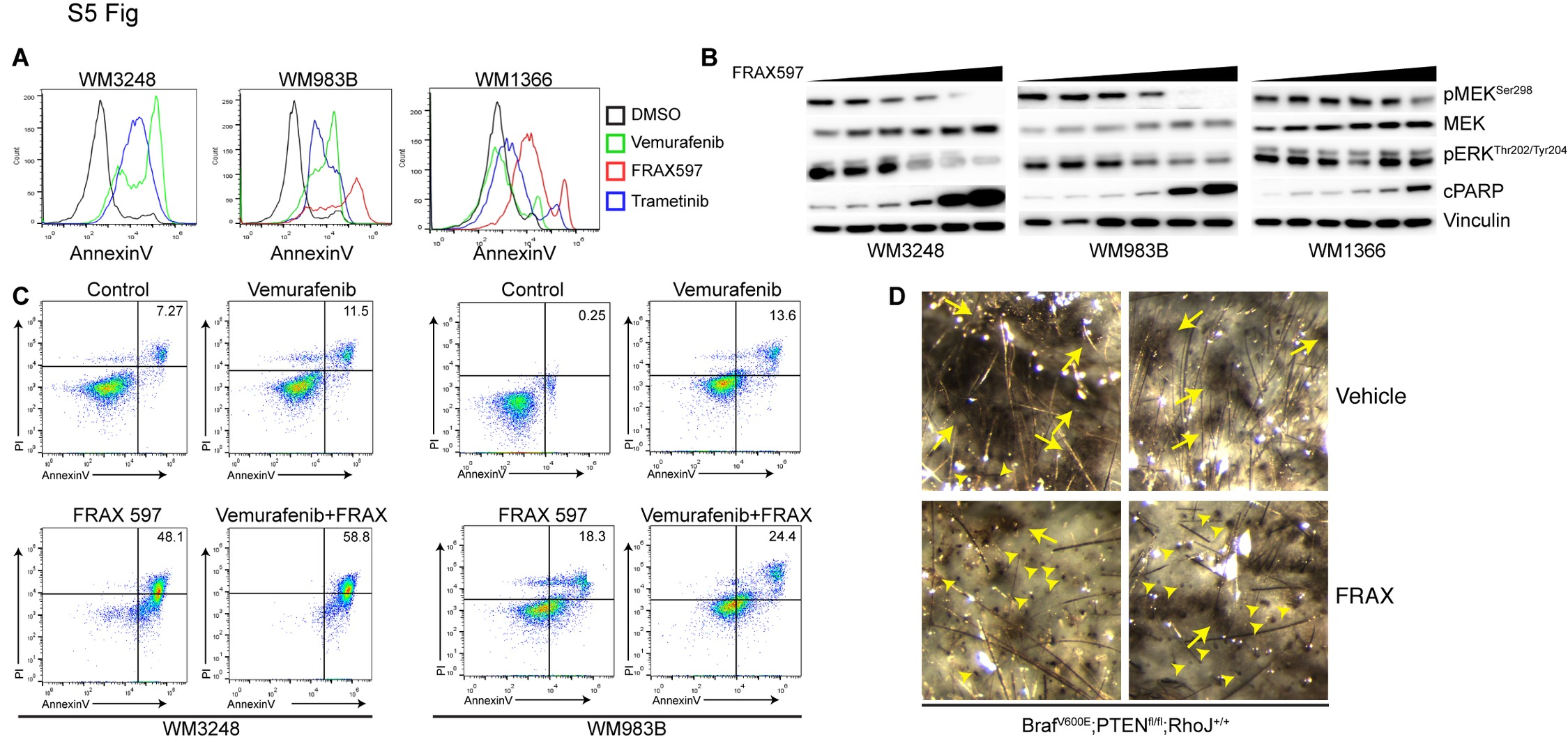

Supplement: S5 Fig — (A) Melanoma cells undergo apoptosis with a 72 hour treatment of FRAX597, Vemurafenib, or Trametinib. All of the cells from WM3248 underwent apoptosis when treated with FRAX597 by 72 hours and is not shown in the graph. (B) FRAX597 inhibits Pak1 activation and induces apoptosis in BRAFV600E melanoma cell lines. Melanoma cell lines harboring either BRAFV600E or BRAFWT were treated with increasing concentrations of FRAX597 (0μM, 0.2 μM, 0.5 μM, 1 μM, 2.5 μM, 5 μM) and immunoblotted with the indicated Abs to measure Pak1 activation (pMEKSer298) and apoptosis (cleaved PARP). (C) FRAX597 does not synergize with Vemurafenib. Cells were treated with either FRAX597, Vemurafenib, or both and processed with FACS. (D) Pak inhibition delays tumor formation. Melanoma was induced as described Fig 5F and administered with vehicle or FRAX597 via oral gavage. Skin images were captured using a dissection scope. (TIF) [file pgen.1006913.s005.tif]
